# MRI-based measurement of masseter muscle area: reliability and clinical relevance in acute neck infections

**DOI:** 10.1007/s00330-026-12500-z

**Published:** 2026-04-03

**Authors:** Akseli Elo, Jari-Pekka Vierula, Jaakko Heikkinen, Janne Nurminen, Tatu Happonen, Mikko Nyman, Jarno Velhonoja, Heikki Irjala, Tero Soukka, Kimmo Mattila, Otso Arponen, Aapo Sirén, Jussi Hirvonen

**Affiliations:** 1 Department of Radiology, University of Turku and Turku University Hospital, Turku, Finland; 2 Department of Otorhinolaryngology-Head and Neck Surgery, University of Turku and Turku University Hospital, Turku, Finland; 3 Department of Oral and Maxillofacial Surgery, University of Turku and Turku University Hospital, Turku, Finland; 4 Medical Imaging Center, Department of Radiology, Tampere University and Tampere University Hospital, Tampere, Finland

**Keywords:** Magnetic resonance imaging, Neck, Infections, Masseter muscle, Sarcopenia

## Abstract

**Objectives:**

The role of low muscle mass in acute neck infections is underexplored. This study aimed to validate the reliability of MRI-based masseter muscle area (MMA) measurements, derive normative age-related data in a diverse patient cohort, and evaluate the association between MMA measurements and infection severity and clinical outcomes in patients with acute neck infections.

**Materials and methods:**

In this retrospective single-center study, 526 patients with clinically confirmed acute neck infections who underwent emergency neck MRI had bilateral MMA measured on T2-weighted Dixon images at the mandibular foramen level. MMA was normalized to height squared (MMA/h^2^). Interobserver reliability of MMA was assessed between two readers using intraclass correlation coefficients (ICC). Among adults, associations with infection severity markers and clinical outcomes were examined using univariable and multivariable tests.

**Results:**

The interobserver agreement for MMA was excellent (ICC = 0.991). MMA/h^2^ declined significantly with age (r = −0.21, *p* < 0.01). In adults, MMA/h^2^ correlated negatively with age, maximal abscess diameter, and length of hospital stay (LOS; *p* < 0.01 for all); patients with abscesses had lower MMA/h^2^ than those without (*p* = 0.002). Multivariable analysis confirmed that MMA/h^2^ independently predicted abscess diameter after adjustment for age and C-reactive protein (CRP) (*p* = 0.001), but not LOS (*p* = 0.136).

**Conclusion:**

Opportunistic MRI-based MMA measurements are highly reliable and associated with age, LOS, and abscess presence and size in acute neck infections. Our findings suggest a link between low masseter muscle mass and disease severity in patients with acute neck infections.

**Key Points:**

***Question*** *Low muscle mass associates with adverse outcomes in various diseases, yet its prognostic role in acute neck infections remains underexplored.*

***Findings*** *MRI-based masseter muscle area measurements showed excellent reliability, declined with age, and predicted larger abscess diameter and longer hospital stay in adult patients.*

***Clinical relevance*** *Opportunistic MRI measurement of masseter muscle area is reliable and clinically associated with abscess size and length of hospital stay in patients with acute neck infections, providing clinically relevant information and supporting early risk stratification*.

**Graphical Abstract:**

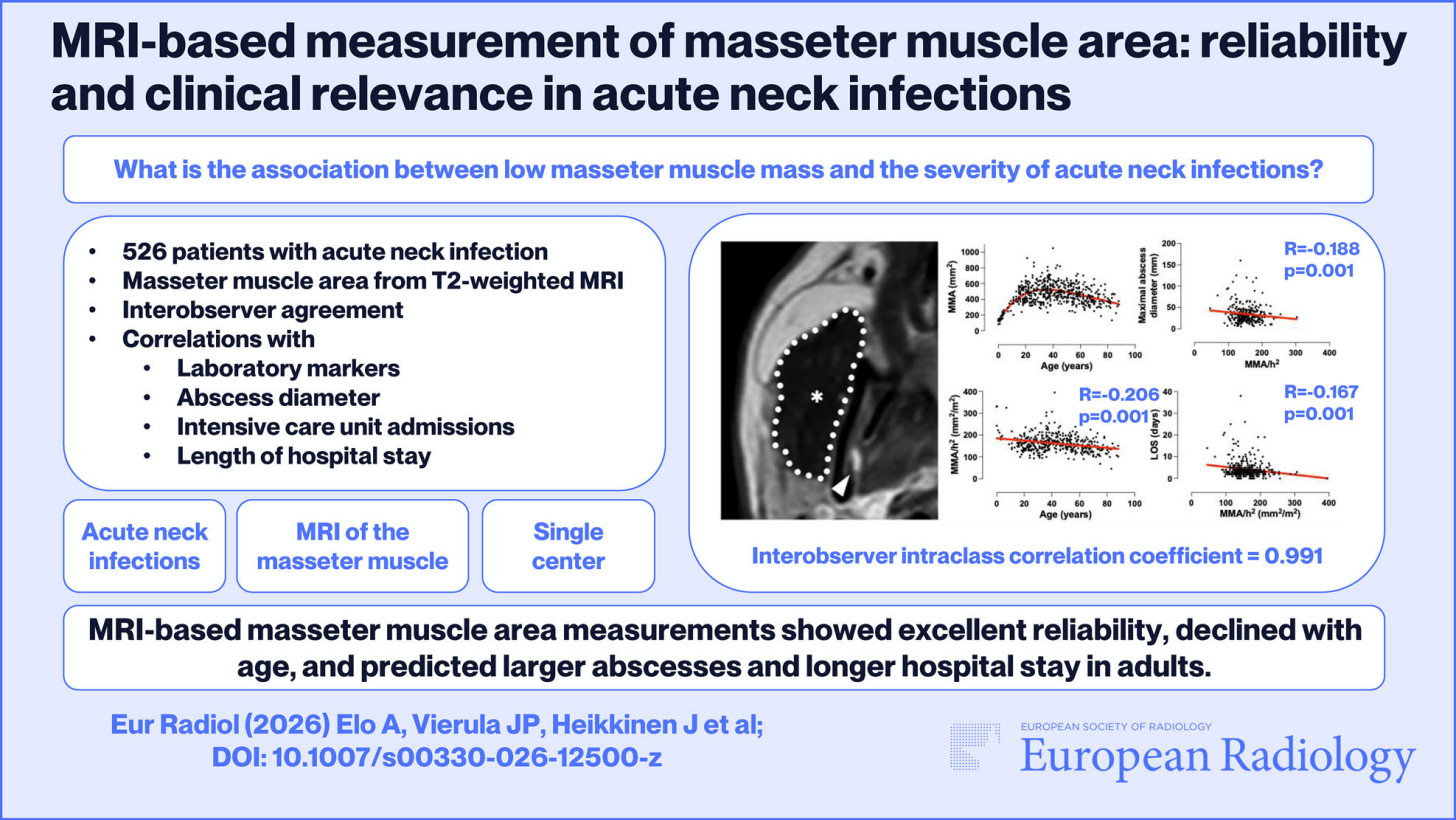

## Introduction

Acute neck infections can rapidly progress to life-threatening complications such as airway obstruction and mediastinitis [1]. Cross-sectional imaging, using CT or MRI, plays a crucial role in managing these patients [2, 3]. The primary clinical value of this imaging is to delineate the extent of infection, identify drainable abscesses and potential complications, and evaluate the involvement of deep fascial spaces, thereby guiding timely interventions such as surgical drainage. CT is most widely used due to its speed and widespread availability for emergency settings, while MRI has a nascent role due to its superior soft-tissue contrast [2, 3].

Cross-sectional medical imaging enables assessment of patients’ muscle status, including muscle mass and quality [4]. Sarcopenia, defined as the progressive loss of muscle mass and strength, is associated with adverse outcomes such as mortality [5, 6]. Research on opportunistic assessments of muscle mass has primarily used abdominal CT scans by measuring one or multiple muscles at the level of the 3rd lumbar vertebra [4]. However, recent studies suggest that masseter muscle area (MMA) and its stature-corrected index (MMA/h^2^) can serve as a regional marker of overall muscle mass [7–11] and may correlate with adverse clinical outcomes across various patient populations [12–15]. While radiologically assessed muscle mass has been widely studied in chronic diseases [16–18], its prognostic value remains unexplored in the context of acute neck infections.

Previously, we have used MRI to image patients with acute neck infections, noting significant variability in disease severity and clinical outcomes [19]. Specifically, patients with large abscesses and widespread edema on imaging tend to have a prolonged course of illness. Yet, the predictive value of these imaging biomarkers is limited, suggesting that additional patient-specific factors are at play. Therefore, we hypothesized that the opportunistic identification of decreased muscle mass could offer prognostic value beyond conventional laboratory and imaging markers in patients with acute neck infections. Our large, real-world cohort, comprising individuals with varying ages and clinical characteristics, enables us to examine the significance of muscle mass in a heterogeneous population across a wide age range. This differs from most sarcopenia research, which primarily focuses on elderly populations [20]. Thus, the present study has three aims. We aimed to assess the reliability of MRI-based MMA measurements, establish normative data for different ages, and examine the association between MMA and markers of infection severity and clinical outcomes in adult patients. Specifically, we hypothesized that low masseter muscle mass would be associated with high disease severity and unfavorable outcomes.

## Materials and methods

### Patients

**Fig. 1:**
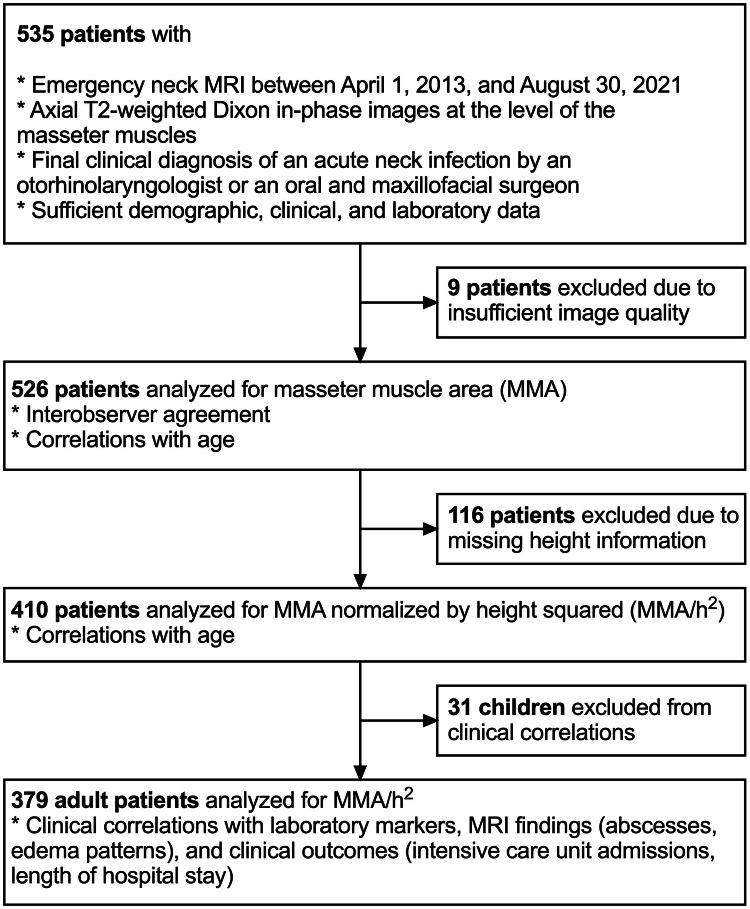
Study flowchart

The requirement for patient consent was waived due to the study’s retrospective nature. The inclusion criteria were: (1) emergency neck MRI between April 1, 2013, and August 30, 2021 due to suspected acute neck infection; (2) emergency MRI evidence of infection: high signal of fat-suppressed T2-weighted Dixon images suggesting edema, or high signal of fat-suppressed post-contrast T1-weighted Dixon images suggesting abnormal tissue enhancement; (3) axial T2-weighted Dixon in-phase images at the level of the masseter muscles; (4) final clinical diagnosis of an acute neck infection by an otorhinolaryngologist or an oral and maxillofacial surgeon; (5) in patients with abscesses, surgical proof of purulence; and (6) sufficient demographic, clinical, and laboratory data. An exclusion criterion was insufficient image quality as judged by a fellowship-trained head and neck radiologist (J.Hi.). Patients aged ≤ 17 years were excluded from the correlation analysis of MMA and infection severity because muscle mass in children and adolescents is actively growing, which could confound associations with disease severity. However, pediatric patients were included in the reliability assessment and in the age-related normative data analysis to provide a comprehensive reference range across the lifespan. The study flowchart is provided in Fig. 1.

Patients were scanned using a Philips Ingenia 3-T system with a dS HeadNeckSpine coil configuration (Philips Healthcare) and a gadolinium-based intravenous contrast agent (Dotarem; Guerbet). The imaging protocol included T1- and T2-weighted images using the Dixon method, diffusion-weighted imaging (DWI), and T1-weighted Dixon sequences after contrast agent administration. Details of the MRI are provided in the Supplementary Information. From the MRI data, we have previously measured the maximal abscess diameter and recorded the following reactive edema patterns, associated with a severe course of illness: retropharyngeal edema (RPE) and mediastinal edema (ME) [19]. We defined an abscess as an abnormal T2-hyperintense collection with a low apparent diffusion coefficient (ADC), surrounded by abnormal tissue enhancement on post-contrast T1-weighted images, and with no enhancement in the center. The maximal abscess diameter was set to 0 in patients with no abscesses to maintain the maximum sample size in the multivariable models. RPE was defined as an area of a hyperintense signal of at least 2 mm in anteroposterior thickness between the prevertebral muscles posteriorly and the superior pharyngeal constrictor muscle anteriorly in at least two consecutive axial fat-suppressed T2-weighted Dixon images [19]. ME was defined as an area of a hyperintense signal in the soft tissues in axial fat-suppressed T2-weighted Dixon images at or below the level of the thoracic inlet, using the superior border of the manubrium sterni or the first thoracic vertebra as the superior border [19].

The following clinical and laboratory information was recorded for this study: age (years), sex (male/female), C-reactive protein (CRP, mg/L), white blood cell count (WBC, × 10^9^/L), admission to the intensive care unit (ICU, yes/no), and the length of hospital stay (LOS). The decision to admit the patient to the ICU was made by the clinicians treating the patient, based on an overall assessment of the patient. Demographic characteristics, laboratory values, clinical outcomes, and proportions of imaging findings consistent with abscess, RPE, and ME are presented in Table 1.

**Table 1:** Patient characteristics

Characteristics	Patients with MMA (*n* = 526)	Patients with MMA/h^2^ (*n* = 410)
Age (years) mean, (SD), range (median)	40.8 (20.7), 0–88 (38)	43.3 (19.6), 0–88 (42)
Female, *n* (%)	227 (43.2)	179 (43.7)
Pediatric*, *n* (%)	64 (12.2)	31 (7.6)
Odontogenic infection, *n* (%)	146 (28)	115 (28)
Pharyngotonsillar infection, *n* (%)	167 (32)	136 (33)
CRP (mg/L) median (IQR), range	109 (112), 1–555	112 (119), 1–555
WBC (× 10^9^/L) mean, (SD), range (median)	13.6 (5.4), 1.9–41.5 (12.9)	13.6 (5.1), 1.9–37.0 (12.9)
Length of hospital stay (days) mean (SD), range (median)	4.1 (3.0), 0–38 (3)	4.2 (3.9), 0–38 (3)
Abscess, *n* (%)	370 (70.3)	296 (72.2)
RPE, *n* (%)	272 (51.7)	219 (53.4)
ME, *n* (%)	130 (24.7)	114 (27.8)
MMA (mm^2^) mean (SD), range (median)	458.8 (134.6), 82.5–1050.0 (456.7)	468.6 (125.7), 82.5–1050.0 (462.5)
MMA/h^2^ (mm^2^/m^2^)	N.A.	161.4 (39.9), 45.6–395.1 (156.2)

*SD* standard deviation, *CRP* C-reactive protein, *WBC* white blood cell count, *RPE* retropharyngeal edema, *ME* mediastinal edema, *MMA* masseter muscle area, *MMA/h*^*2*^ masseter muscle area (mm^2^) divided by patients’ heights (m) squared

* Aged ≤ 17 years

### Masseter muscle area measurements

**Fig. 2:**
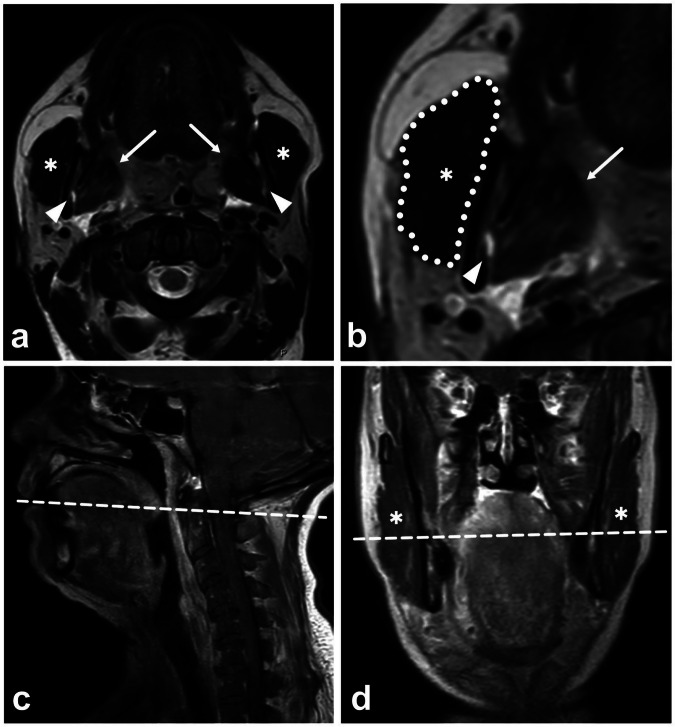
MR images of a 37-year-old female with deep neck infection. Axial T2-weighted (**a**), axial T2-weighted, inset from **a** (**b**), sagittal T1-weighted with gadolinium-based contrast media (**c**), coronal T1-weighted with gadolinium-based contrast media (**d**). Masseter muscles are denoted with white asterisks (**a**, **b**, **d**). The masseter muscle area (area inside dotted white line, **b**) was measured at the level of the mandibular foramen (white arrowheads, **a**, **b**). MRI’s sensitivity in depicting muscle edema is illustrated in the medial pterygoid muscles (white arrows). The right medial pterygoid is edematous due to infection, while the left one is normal (**a**, **b**). The level of axial image (**a**, **b**) is demonstrated with white dashed lines in sagittal and coronal planes (**c**, **d**)

MMA regions of interest were manually delineated on axial T2-weighted in-phase Dixon images using Philips Picture Archiving and Communication System (Philips Healthcare) for the right and left masseter muscles at the level of the mandibular foramen according to a previously published method, following the outline of the muscle [8] (Fig. 2). Tilt was not adjusted, but the measurement level was independently selected on each side to correspond to the mandibular foramen in cases of obliqueness. The right and left areas were averaged to yield a single measurement per patient. Muscles with edema evident on T2-weighted Dixon water-only images, e.g., due to odontogenic neck infections, were excluded to avoid overestimation of muscle mass (*n* = 68, 13% of the final sample), and single values from the unaffected muscle were accepted as the final MMA. Assessment of muscle edema was performed independently by a fellowship-trained head and neck radiologist (J.Hi.) on T2-weighted Dixon water-only images before muscle area measurements. Cases with evident masseter muscle edema were flagged, and for these patients, only the unaffected contralateral muscle was measured. Reader 1, a medical student (A.E.), performed the measurements for all patients after supervised training. To measure interobserver agreement, a random sample of 30 patients was independently evaluated by Reader 2, a fellowship-trained head and neck radiologist (J.Hi.). To scale the measurement according to body proportions, MMA (mm^2^) was normalized for stature by dividing it by patient height squared (m^2^), yielding a scaled value MMA/h^2^, as previously done [7–9].

### Statistical analyses

This study employed an exploratory design to examine associations between MMA/h^2^ and multiple markers of infection severity and clinical outcomes. We examined two imaging markers (abscess presence/size, edema patterns), two laboratory markers (CRP, WBC), and two clinical outcome measures (ICU admissions, LOS). Given the exploratory nature, no single primary endpoint was pre-specified; instead, we sought to comprehensively characterize the relationship between muscle mass and various aspects of acute neck infection severity.

Interobserver agreement was assessed using mean absolute variability (VAR) and intraclass correlation coefficient (ICC). A sample size of 30 patients was selected based on established guidelines [21]. ICC represents the proportion of total variance attributable to between-patient variance relative to interobserver variance. VAR was defined as the mean of absolute differences between MMA measurements from both readers divided by the mean value: |Reader 1 − Reader 2| / 0.5 × (Reader 1 + Reader 2). ICC was interpreted as follows: < 0.5, poor; 0.5–0.75, moderate; 0.75–0.9, good; > 0.9, excellent.

At the univariable level, we analyzed associations between MMA or MMA/h^2^ and nominal variables using *t*-tests, and between MMA or MMA/h^2^ and continuous variables using Pearson correlation coefficients.

Multivariable linear regression models were constructed with either the maximal abscess diameter or the LOS as the dependent variable. Independent variables were selected based on clinical relevance and prior research in this patient population [19]. For the abscess diameter model, predictors included MMA/h^2^, age, and CRP. For the LOS model, predictors included MMA/h^2^, age, CRP, and maximal abscess diameter. All predictors were entered into the model simultaneously (forced entry method). Clinical correlations were conducted only in adult patients (aged 18 years or older) due to the potentially confounding impact of normally growing muscle mass in children. Analyses were performed using listwise deletion of cases with missing values on any variable in the model. Missing data were minimal (< 1%).

Model assumptions were verified using both visual diagnostics and formal statistical tests. Residual plots confirmed linearity, with no systematic patterns observed. Shapiro–Wilk tests for normality of residuals showed statistically significant deviations (Model 1: W = 0.95, *p* < 0.001; Model 2: W = 0.73, *p* < 0.001); however, Q-Q plots demonstrated that residuals followed approximately normal distributions. Breusch-Pagan tests detected heteroscedasticity (Model 1: BP = 55.7, *p* < 0.001; Model 2: BP = 13.9, *p* = 0.008), but visual inspection of scale-location plots confirmed acceptable variance patterns. Multicollinearity was absent (all variance inflation factors (VIF) < 1.3).

Continuous values are reported as means ± standard deviations (SD) and categorical variables as crude numbers and percentages unless otherwise stated. Standard errors (SE) are provided for regression coefficients. Statistical analyses were performed using R (version 4.3.3, R Foundation for Statistical Computing) and packages *car* (VIF), *lmtest* (heteroscedasticity), and *tidyverse* (data manipulation). *p*-values < 0.05 were considered statistically significant.

## Results

The total study sample included 526 patients (Table 1, Fig. 1). Height data were available for 410 patients. The mean age was 40.8 ± 20.7 (SD) years (range 0–88). The mean MMA was 458.8 ± 134.6 mm^2^ (range 82.5–1050). MMA and MMA/h^2^ values are reported in Table 1. MMA measurements were highly similar between the readers; in the test sample, the average MMA was 445.2 mm^2^ for Reader 1 and 445.3 mm^2^ for Reader 2. The average VAR was 1.8%, and the ICC was 0.991 (95% confidence interval 0.981–0.996, *p* < 0.001), indicating excellent interobserver agreement.

**Fig. 3:**
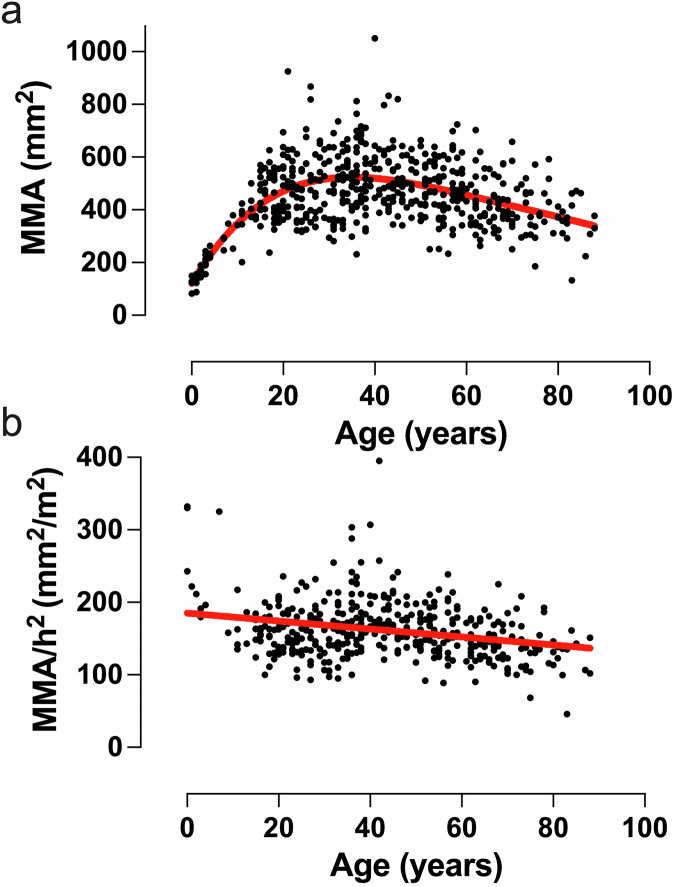
MMA (**a**) and MMA/h^2^ (**b**) according to age in the whole study sample. For visualization purposes, MMA data were fitted with a quadratic polynomial function (**a**) and MMA/h^2^ data with a linear regression analysis

Masseter muscle area values increased with age until adulthood and declined thereafter (Fig. 3a). The mean MMA/h^2^ was 161.4 ± 39.9 mm^2^/m^2^, ranging between 45.6–395.1 mm^2^/m^2^. In patients aged 18 or older, MMA/h^2^ demonstrated a statistically significant negative correlation with age (Pearson’s r = −0.206, *p* < 0.01), indicating a progressive decline in masseter muscle size with increasing age (Fig. 3b).

**Fig. 4:**
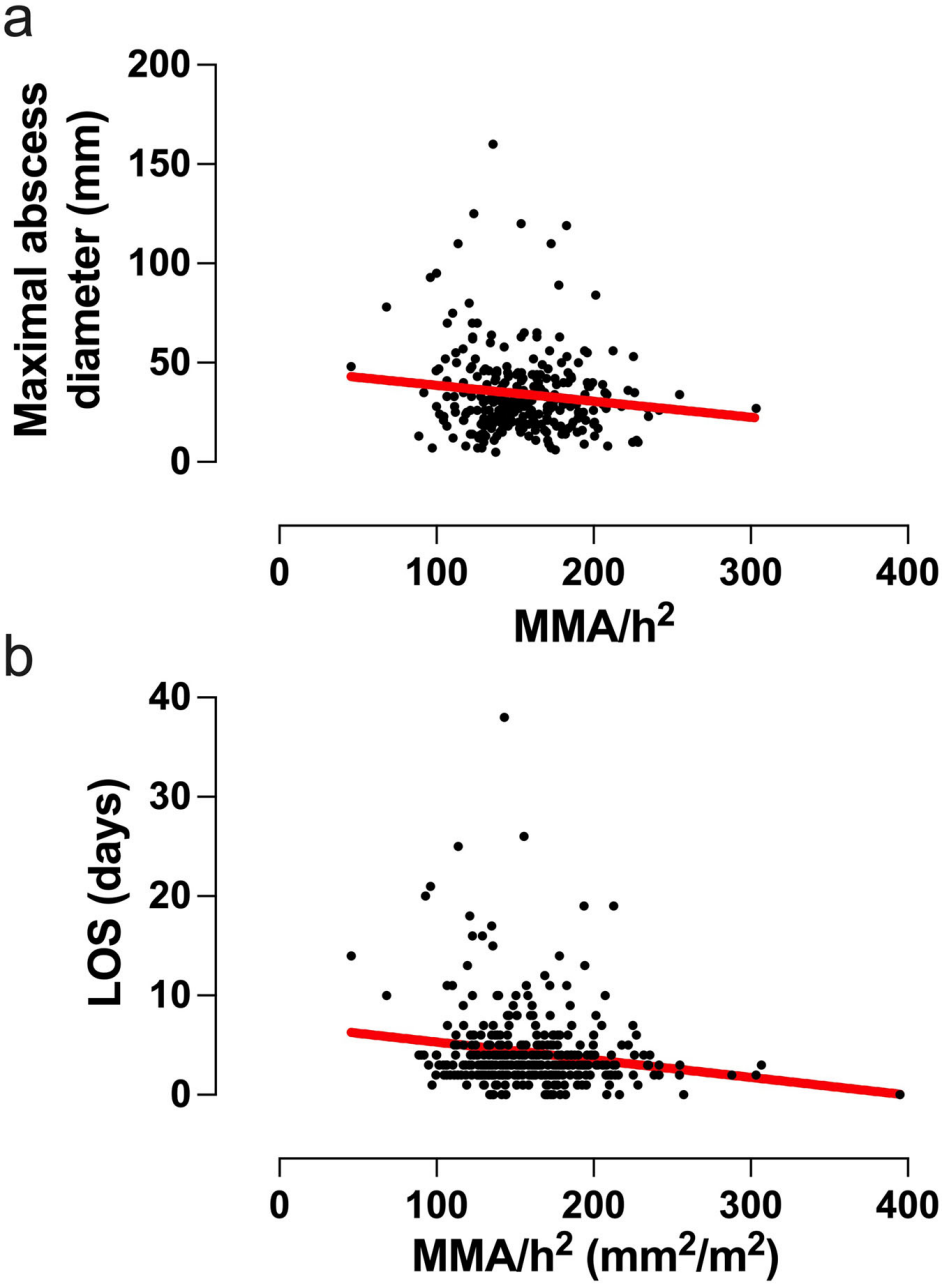
Associations between MMA/h^2^ and maximal abscess diameter (**a**) and LOS (**b**) in adult patients. The red lines represent linear regression fits

**Fig. 5:**
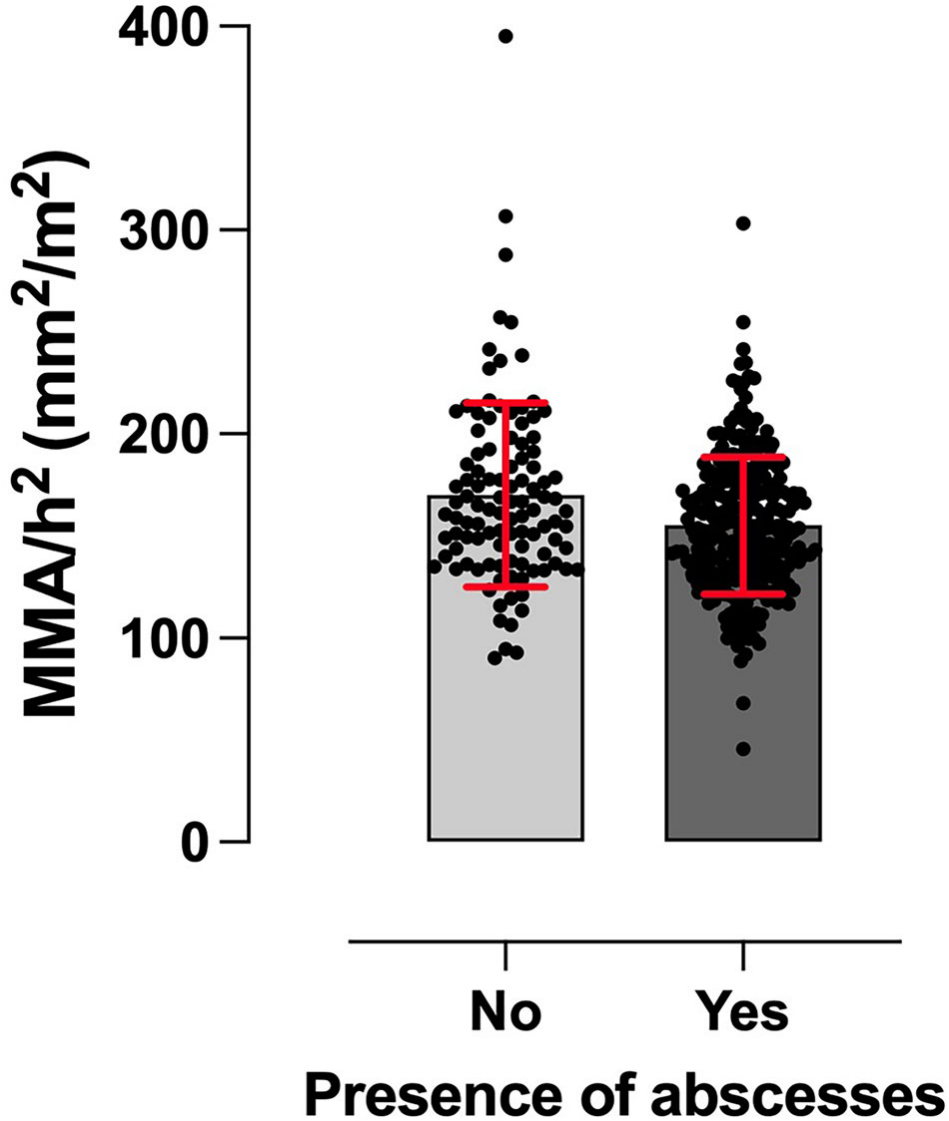
MMA/h^2^ in patients with and without abscesses. Box height represents means, and bars represent standard deviations

In univariable analyses considering measures of infection severity among patients aged 18 or older, MMA/h^2^ correlated negatively with age (r = −0.206, *p* = 0.001), maximal abscess diameter (r = −0.188, *p* = 0.001), and LOS (r = −0.167, *p* = 0.001) (Fig. 4). In contrast, MMA/h^2^ did not correlate with CRP (*p* = 0.113) or WBC (*p* = 0.291). Patients with abscesses had lower MMA/h^2^ than those without (*p* = 0.002) (Fig. 5). A *t*-test showed no significant difference in MMA/h^2^ between patients who required ICU admission and those who did not (*p* = 0.111). Similarly, *t*-tests comparing MMA/h^2^ in patients with or without mediastinal edema or retropharyngeal edema showed no significant differences (*p* = 0.204 and *p* = 0.278, respectively).

In the multivariable linear regression model predicting maximal abscess diameter (376 patients after listwise deletion), MMA/h^2^ independently predicted larger abscess size (β = −0.098, SE = 0.030, *p* = 0.001). CRP was also a significant predictor (β = 0.111, SE = 0.013, *p* < 0.001), while age was not (β = −0.039, SE = 0.063, *p* = 0.534). The overall model was significant (F(3,372) = 30.87, *p* < 0.001, *R*^2^ = 0.199). All VIFs were < 1.1, confirming the absence of multicollinearity.

In the multivariable model predicting LOS (374 patients after listwise deletion), MMA/h^2^ did not independently predict LOS after adjusting for age, CRP, and abscess diameter (β = −0.008, SE = 0.005, *p* = 0.136). Significant predictors included age (β = 0.036, SE = 0.011, *p* < 0.001), CRP (β = 0.013, SE = 0.002, *p* < 0.001), and maximal abscess diameter (β = 0.031, SE = 0.009, *p* < 0.001). The overall model was significant (F(4,369) = 22.79, *p* < 0.001, *R*^2^ = 0.198). All VIF values were < 1.3, confirming the absence of multicollinearity.

## Discussion

In this study, we measured the masseter muscle area on routine emergency MRIs to explore the association between low muscle mass and clinical features of acute neck infections. The measurements were highly reliable with excellent interobserver agreement. MMA/h^2^ negatively correlated with age, presence and maximal diameter of abscesses, and LOS. Even after controlling for age and CRP, MMA/h^2^ was independently associated with maximal abscess diameter, indicating potential prognostic value for infection severity. These exploratory results suggest the potential utility of opportunistic muscle mass measurements from emergency MR images and warrant further research to evaluate their broader application in acute infectious diseases.

Regarding the planned endpoints of our study, MMA/h^2^ showed univariable associations with LOS but no relationship with ICU admission or retropharyngeal/mediastinal edema. These heterogeneous results indicate that low muscle mass may preferentially be associated with the severity of localized infections (e.g., abscess formation) rather than systemic complications. Such mixed findings are valuable, as they highlight the nuanced role of low muscle mass in acute infections, similar to observations in geriatric cohorts, where masseter area correlates with overall sarcopenia but not dysphagia [22], or in head and neck cancer, where masseter indices predict survival variably [14]. This underscores the need for prospective studies to assess context-specific prognostic utility.

Our findings partly align with recent evidence linking low muscle mass to higher severity in acute neck infections [23]. Kikuta et al demonstrated that CT-assessed muscle mass at the C^3^ level mathematically converted to lumbar measurements independently predicted infection severity in odontogenic infections and also showed a weak but significant, independent correlation with prolonged LOS. The differences in LOS findings may be attributed to variations in patient populations, inclusion and exclusion criteria, management strategies, and other factors. However, these similar findings regarding the association between low muscle mass and infection severity highlight the potential of opportunistic muscle mass assessments for predicting the severity of acute neck infections.

The association between lower MMA/h^2^ and infection severity, particularly abscess size, is likely multifactorial. On the systemic level, lower muscle mass may indicate frailty, weakened immune function, and malnutrition [5, 24, 25]. Specifically, sarcopenic muscle exhibits heightened proteolysis driven by proinflammatory cytokines such as tumor necrosis factor α and interleukin 6, which accelerate muscle wasting [26, 27]. This immunosenescence-muscle axis positions low MMA/h^2^ as a proxy for systemic vulnerability in acute inflammatory states. As a masticatory muscle, the masseter may also indicate nutritional status through its chewing function [10]; it is plausible that a reduced masseter cross-sectional area correlates with diminished masticatory efficiency, leading to poor nutrition and increased susceptibility to infection. In addition to these systemic mechanisms, local inflammatory response and anatomical factors, such as those related to fasciae connecting anatomical compartments, may also contribute to abscess size. However, this seems unlikely, as the abscess was not always immediately adjacent to the masseter muscles. Kikuta et al also considered the possibility of reverse causality, where infection and local inflammation cause acute muscle loss [23]. However, even though muscle loss can be rapid [28, 29], acute inflammation might skew the results in the opposite direction through muscle swelling. Taken together, our study design was cross-sectional, and we therefore cannot make causal inferences; longitudinal studies are needed to unravel these dynamics. One potential clinical implication is the importance of prioritizing nutritional support in patients with acute neck infections.

This study has several strengths, including a large sample size of 526 patients drawn from a heterogeneous, real-world population. Despite the inherent subjectivity of manual masseter muscle segmentation, interobserver agreement was excellent (ICC = 0.991), underscoring high reliability and feasibility for clinical adoption. A notable advantage lies in the use of T2-weighted, fat-saturated Dixon MRI sequences, which enabled precise identification and unilateral exclusion of muscles exhibiting intramuscular edema in 13% of cases (mostly odontogenic infections), thereby minimizing measurement bias and leveraging the superior soft-tissue contrast of MRI over CT. Nevertheless, some limitations need to be considered. Our study was retrospective, which may introduce bias. Importantly, our study was conducted at a single tertiary care center using a single MRI scanner, which may limit generalizability to other healthcare settings, patient characteristics, MRI vendors, field strengths, or sequence protocols. Multicentre validation studies are needed to confirm the generalizability of our findings. Broader applicability may be restricted by the lower availability of emergency MRI in other centers. Muscle mass was assessed solely via imaging without complementary functional tests. Additionally, measurements were performed on axial slices that were not always perpendicular to the long axis of the muscle, which could introduce a minor volumetric bias; however, this approach prioritized rapid assessments. Volumetric quantification might offer finer granularity, but was not done here due to time constraints. In the future, prospective multicentre studies incorporating automated artificial intelligence-based segmentation could address these limitations. Also, future studies incorporating quantitative muscle composition metrics, such as fat fraction derived from Dixon imaging, may provide complementary information beyond muscle area alone and could enhance risk stratification. Finally, our study lacked a healthy control group without acute neck infections. We did not systematically evaluate for temporomandibular joint disorders or bruxism, both of which can influence masseter muscle size.

In conclusion, MRI-based MMA measurements showed high reliability, as suggested by excellent interobserver agreement. Normalized MMA/h^2^ declines with age, correlates negatively with LOS, and independently predicts maximal abscess diameter, suggesting its utility as a prognostic biomarker in acute neck infections.

## Supplementary information

ELECTRONIC SUPPLEMENTARY MATERIAL

## Abbreviations

BP: Breusch-Pagan statistic
CRP: C-reactive protein
ICC: Intraclass correlation coefficient
ICU: Intensive care unit
LOS: Length of hospital stay
ME: Mediastinal edema
MMA: Masseter muscle area
MMA/h^2^: Masseter muscle area normalized to height squared
RPE: Retropharyngeal edema
SD: Standard deviation
SE: Standard error
VAR: Mean absolute variability
VIF: Variance inflation factor
WBC: White blood cell count
