# Supplementary material for: MRI-based measurement of masseter muscle area: reliability and clinical relevance in acute neck infections
Source: Eur Radiol. 2026 Apr 3;36(8):6365–75. doi: 10.1007/s00330-026-12500-z (PMC13341821; doi:10.1007/s00330-026-12500-z)
Supplement: Supplementary file 1 — ELECTRONIC SUPPLEMENTARY MATERIAL [file 330_2026_12500_MOESM1_ESM.pdf]

MRI-based measurement of masseter muscle area: reliability and clinical relevance in acute neck infections

ELECTRONIC SUPPLEMENTARY MATERIAL

Eur Radiol (2026) Elo A, Vierula JP, Heikkinen J et al.

Table S1. MRI protocol for acute neck infections

Sequence	Parameters	Acquisition time
T2 Dixon axial	Slice thickness 4 mm, TE=100 ms, TR=3021 ms, flip angle 90°	3 min 46 s
DWI axial	Slice thickness 4 mm, TE=87 ms, TR=3981 ms, b-value 1000 s/mm, flip angle 90°	48 s
T2 Dixon coronal	Slice thickness 3.5 mm, TE=80 ms, TR=3210 ms, flip angle 90°	5 min 14 s
T1 TSE axial	Slice thickness 4 mm, TE=10 ms, TR=641 ms, flip angle 90°	4 min 24 s
T1 Dixon axial post-contrast	Slice thickness 4 mm, TE=7 ms, TR=634 ms, flip angle 90°	3 min 29 s
T1 Dixon coronal post-contrast	Slice thickness 3.5 mm, TE=14 ms, TR=560 ms, flip angle 90°	3 min 8 s
T1 Dixon sagittal post-contrast	Slice thickness 3 mm, TE=14 ms, TR=630 ms, flip angle 90°	3 min 6 s
Total scan time		23 min 55 s
